# Supplementary material for: Individual and city-level variations in heat-related road traffic deaths in Latin America
Source: medRxiv. 2025 Sep 7:2025.09.05.25334734. Preprint. [Version 1] doi: 10.1101/2025.09.05.25334734 (PMC12425034; doi:10.1101/2025.09.05.25334734)
Supplement: 1 [file NIHPP2025.09.05.25334734V1-supplement-1.pdf]

medRxiv preprint doi: https://doi.org/10.1101/2025.09.05.25334734; this version posted September 7, 2025. The copyright holder for this preprint (which was not certified by peer review) is the author/funder, who has granted medRxiv a license to display the preprint in perpetuity.
It is made available under a CC-BY 4.0 International license .

Supplementary Information

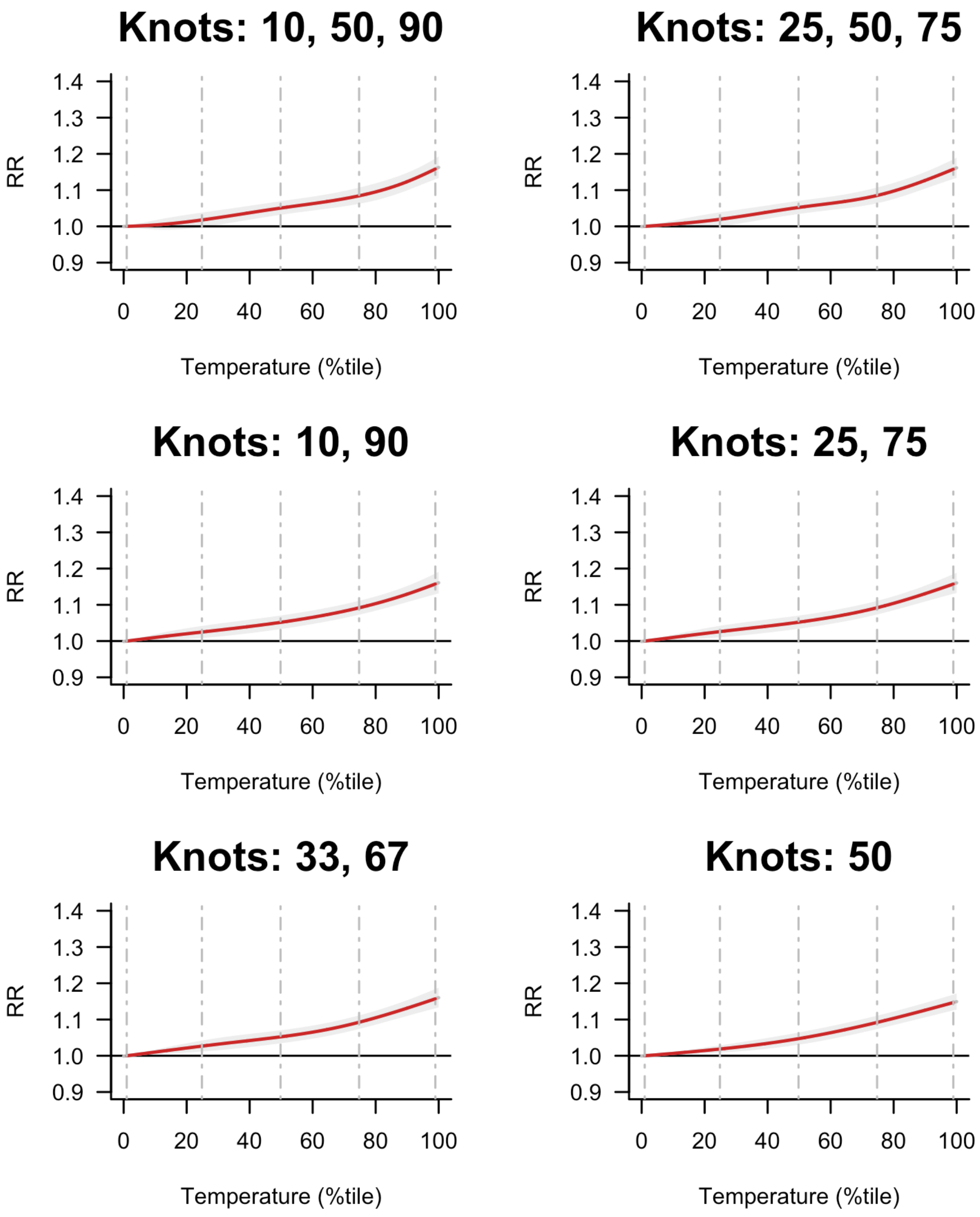

Supplementary Fig. 1. Sensitivity analyses testing different knot specifications. *Dashed vertical lines indicate temperature percentiles: 1st, 25th, 50th, 75th, and 99th from left to right. Shading in each association curve represents the 95% confidence interval.*

medRxiv preprint doi: https://doi.org/10.1101/2025.09.05.25334734; this version posted September 7, 2025. The copyright holder for this preprint
(which was not certified by peer review) is the author/funder, who has granted medRxiv a license to display the preprint in perpetuity.
It is made available under a CC-BY 4.0 International license .

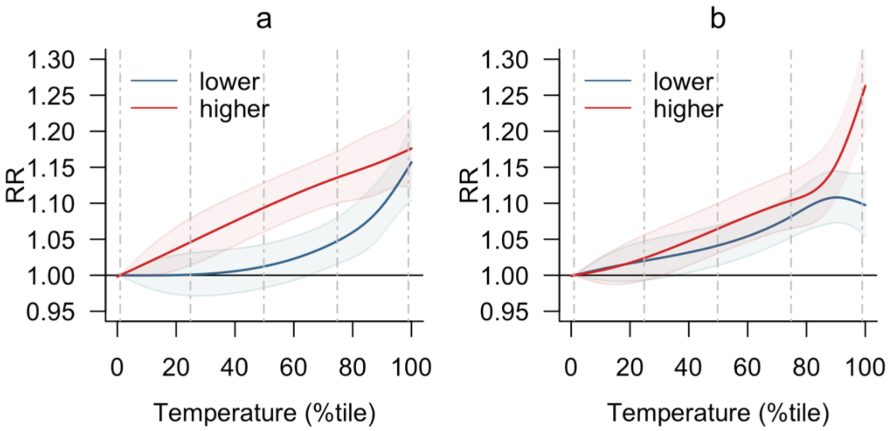

Supplementary Fig. 2. Effect modification by summaries of city-level temperature profiles.
(a) Mean temperature. (b) Standard deviation of temperature. High (red) and low (blue) indicate cities at the 90th percentile (higher values) or 10th percentile (lower values) of respective temperature summary. Dashed vertical lines indicate temperature percentiles: 1st, 25th, 50th, 75th, and 99th from left to right. Shading in each association curve represents the 95% confidence interval.

442
